# Globally, GDP Per Capita Correlates Strongly with Rates of Bystander CPR

**DOI:** 10.5334/aogh.3624

**Published:** 2022-05-24

**Authors:** Aditya Shekhar, Jagat Narula

**Affiliations:** 1 Harvard Medical School, US; 2 Icahn School of Medicine at Mount Sinai, US

**Keywords:** Cardiac arrest, bystander CPR, global health, disparities, development

## Abstract

**Introduction:**

Bystander CPR is vital in improving outcomes for out-of-hospital cardiac arrest. There has been ample literature describing disparities in bystander CPR within specific countries, such as the United States, Australia, and the Netherlands. However, there has not been significant literature describing such disparities between countries.

**Methods:**

We examined various studies published between 2000 and 2021 that reported rates of bystander CPR in various countries. These bystander CPR rates were correlated with the GDP per capita of that country during the time the study was conducted. The correlation between GDP per capita and rates of bystander CPR was assessed.

**Results:**

A total of 29 studies in 35 communities across 25 countries were examined. Reported rates of bystander CPR ranged from 1.3% to 72%. From this, a strong and significant correlation between GDP per capita and rates of bystander CPR was apparent; 0.772 (p < .01), r^2^ = 0.596.

**Conclusions:**

GDP per capita can be thought of as a composite endpoint that takes into account various aspects of a country’s social and economic well-being. Socioeconomically-advantaged communities likely have a better ability to provide CPR education to community members, and our findings mirror localized analyses comparing socioeconomic status and rates of bystander CPR. Future studies should continue to elucidate transnational disparities in cardiac arrest, and efforts should be directed at providing CPR education to communities with low rates of bystander CPR; low-and-middle-income countries may represent attractive targets for such interventions. However, it may be possible that rates of bystander CPR may not improve unless significant upstream improvements to socioeconomic factors take place.

## Background

Bystanders can fulfill an important role in the care of out-of-hospital arrest (OHCA) by initiating cardiopulmonary resuscitation (CPR) prior to the onset of permanent injury [1]. An overwhelming consensus in the literature indicates patients who receive adequate bystander CPR achieve markedly better outcomes than patients who do not [2]. Troublingly, there has been data revealing alarming disparities in bystander CPR across various demographic metrics. For example, several studies report higher neighborhood socioeconomic status is associated with increased rates of bystander CPR [3,4,5]. While there are undoubtedly variations in socioeconomic status within a given country, significant differences also likely exist when comparing nations or regions of the world.

Single studies that have examined multinational rates of bystander CPR mainly have focused on single regions. One study published in 2020 compared cardiac arrests in Victoria, Australia with cardiac arrests in Singapore and found rates of bystander CPR were higher in Australia [6]. Another study published in 2015 used data from the Pan Asian Resuscitation Outcomes Study (PAROS) to report rates of bystander CPR across 7 localities in Asia (Japan, Singapore, South Korea, Malaysia, Taiwan, Thailand, and Dubai) – they found rates of bystander CPR ranged from 10.5% in Dubai to 40.2% in Japan and 40.9% in South Korea [7]. These studies highlight the existence of enormous differences in rates of bystander CPR across various countries.

Further study comparing rates of bystander CPR across borders is certainly warranted. Given prior research examining rates of bystander CPR across socioeconomic lines, it might be worthwhile to compare rates of bystander CPR with country-level indicators of socioeconomic status. Such an analysis comparing bystander CPR rates across various regions would be of significant interest to the global cardiovascular and emergency care communities.

## Methods

We searched for studies published between 2000 to 2021 from various countries where rates of bystander CPR were reported. Rates of bystander CPR within a given country were then correlated with that country’s GDP per capita using data from the World Bank for the year(s) when data were gathered. GDP per capita was reported in US Dollars, and studies in countries or regions where World Bank data was not available were excluded. Due to constraints on reference limits, our search was not exhaustive, and we prioritized showcasing a diversity of countries over including many studies from a single country or region. Our study is meant to provide preliminary support for the existence of transnational disparities in bystander CPR, a topic that has received relatively little examination from the resuscitation community.

A Pearson’s Product-Moment Correlation Coefficient was calculated for the correlation between GDP per capita and rates of bystander CPR [8]. Significance was defined as p < .01. Some studies examined rates of bystander CPR over multiple years. If these studies reported data on a year-to-year basis, only the final year from the study was included. If these studies did not report data on a year-to-year basis, a median GDP per capita for that country during the study period was calculated. Studies that examined rates of bystander CPR during the COVID-19 pandemic – defined as beginning after the World Health Organization’s (WHO) official declaration on March 11, 2020 – were excluded because reports suggest rates of bystander CPR changed during the pandemic [9,10]. Since all data were publicly available, formal ethics approval was not sought.

## Results

**Figure 1:**
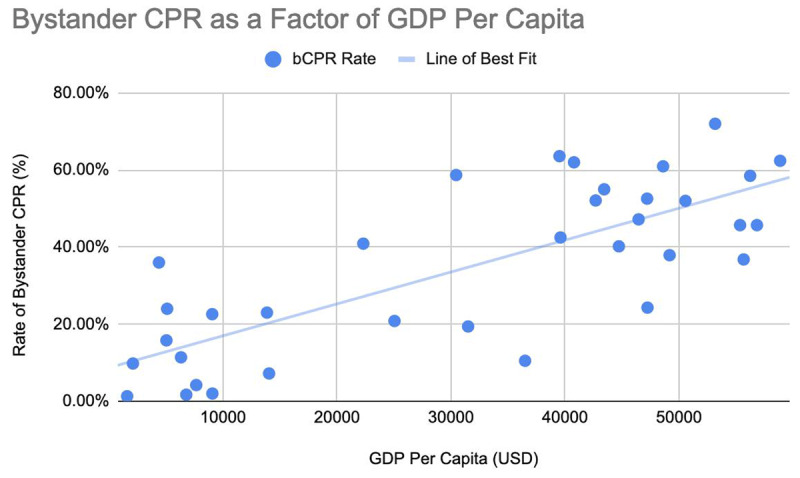
Shows a summary of the 35 communities in 25 different countries examined by the 29 studies we reviewed. Visually, there is a perceptible association between GDP per capita and rates of bystander CPR. Quantitatively, a significant correlation was identified (0.772, p < .01).

Twenty-nine studies were ultimately included [6,7,11,12,13,14,15,16,17,18,19,20,21,22,23,24,25,26,27,28,29,30,31,32,33,34,35,36,37,38]. These 29 studies examined a total of 35 communities in 25 countries, generating a total sample size of 573 818 cardiac arrests. Geographically, 11 studies took place in Asia, 9 took place in Europe, 6 took place in Australasia, 4 took place in North America, 4 took place in the Middle East, and 1 took place in Africa. Rates of bystander CPR varied from 1.3% to 72%. Across all studies, the GDP per capita of a country correlated significantly with rates of bystander CPR (0.772, r^2^ = 0.596, p < .01) (Figure 1).

## Discussion

Our study shows that there is a significant association between GDP per capita and rates of bystander CPR. However, this association certainly goes far beyond merely asserting increases in GDP per capita will coincidentally increase rates of bystander CPR. Rather, GDP per capita appears to be a composite endpoint for a number of socioeconomic and cultural factors that predispose a given population to have higher rates of bystander CPR. This follows similar logic used by localized studies that have reported correlations between neighborhood-level socioeconomic status and rates of bystander CPR. For instance, a community that is socioeconomically-advantaged will likely have more resources to devote to CPR training for community members. This aligns with the idea that socioeconomic status is correlated with health literacy and other studies that have shown links between socioeconomic status and bystander CPR rates [3,4,5,38]. In our example, we consider understanding and being able to perform CPR in an emergency scenario as a form of health literacy. Of course, there certainly could be situations where low-and-middle-income countries are able to achieve high rates of bystander CPR. For instance, a wide-spread cultural emphasis on cardiac or overall health might lead to high rates of bystander CPR independent of socioeconomic factors.

We specifically chose GDP per capita as the marker for socioeconomic status for a number of reasons. First, GDP per capita accounts for different population sizes across countries, allowing for comparisons between countries with differing populations. For this reason, simply examining GDP would not have been as effective. Second, there is relatively reliable GDP per capita data available for all countries examined from a single source (The World Bank). Data for other metrics may not be as readily available. We do recognize that GDP per capita is not a perfect measure of socioeconomic status within a country and that there have been legitimate criticisms regarding GDP per capita as a measure [39]. However, we felt that it best allowed for comparisons between distant countries to be made, and we emphasize its role as a composite endpoint for socioeconomic status.

Our study has a number of limitations that are inevitable with this type of investigation. First, GDP per capita does not take into account within-country differences in socioeconomic status. This is important to mention, since some of the studies examined only reported rates of bystander CPR within a specific region of a country. Unfortunately, data for local-level economic output per capita is not as reliable as whole-country GDP per capita. Second, not all the studies were conducted at the same time. We attempted to rectify this by using GDP per capita data during the time the studies were conducted. An ideal situation would involve examining studies conducted during the same time period, however, we felt that including a wider diversity of studies from a multitude of different countries would be more impactful.

Comparing rates of bystander CPR and other relevant cardiac arrest metrics between countries should still be of great interest in the coming years. The PAROS study is a particularly good example where centers from multiple nations were able to report identical metrics for a given time period [7]. Replicating that with more countries and more centers would be a valuable asset to the literature. Additionally, it would also be interesting to compare economic indicators with other bystander interventions.

## Conclusions

Our data show, across 35 communities in 25 different countries, GDP per capita correlates strongly with rates of bystander CPR. This correlation likely reflects the fact that socioeconomically-advantaged communities are likely better able to train their populations in CPR and emergency care. Our study joins a growing consensus that economic and social well-being correlates with increased health literacy. Lastly, given the importance of bystander CPR, training more individuals in CPR will be vital in reducing the burden of out-of-hospital cardiac arrest, especially in low-and-middle-income countries and regions where rates of bystander CPR are low. However, it may be possible that rates of bystander CPR may not improve unless significant upstream improvements to socioeconomic factors take place.
